# Transcriptome Analysis of Dimethyl Fumarate Inhibiting the Growth of *Aspergillus carbonarius*

**DOI:** 10.3390/toxins17070339

**Published:** 2025-07-04

**Authors:** Siruo Wang, Bowen Tai, Xifan Yu, Erfeng Li, Gang Wang, Jing Jin, Fuguo Xing

**Affiliations:** 1 Key Laboratory of Agro-Products Quality and Safety Control in Storage and Transport Process, Ministry of Agriculture and Rural Affairs, Institute of Food Science and Technology, Chinese Academy of Agricultural Sciences, Beijing 100193, China; wangsiruo163@163.com (S.W.); taibowen@caas.cn (B.T.); 201906022623@zjut.edu.cn (X.Y.); wanggang02@caas.cn (G.W.); jinjing@caas.cn (J.J.); 2 College of Horticulture and Landscape, Tianjin Agricultural University, Tianjin 300384, China; lef143@126.com

**Keywords:** *Aspergillus carbonarius*, dimethyl fumarate, transcriptome, Mycelium growth

## Abstract

*Aspergillus carbonarius* is one of the main pathogens responsible for postharvest diseases in fruits and is also one of the main ochratoxin A-producing strains. It not only causes significant economic losses but also poses a risk to human health. We found an inhibitory effect of dimethyl fumarate fumigation on the growth of *A. carbonarius*. To further explore its antifungal mechanism, this study elucidated the functions of key pathway-related genes through a transcriptomics analysis. A total of 1402 differentially expressed genes (DEGs) were identified, including 987 up-regulated and 415 down-regulated genes. Dimethyl fumarate was found to significantly inhibit the growth of *A. carbonarius* by disrupting cell integrity and obstructing mycelium growth and secondary metabolism. These findings provide a basis for the potential application of dimethyl fumarate in the food industry to inhibit *A. carbonarius* and subsequent ochratoxin A contamination.

## 1. Introduction

*Aspergillus carbonarius* is a significant postharvest pathogen affecting fruits, particularly grapes, leading to rot and the production of ochratoxin A (OTA). It poses a serious threat to fruit quality, contributing to substantial economic losses [1] and health risks [2]. It is the most common pathogen species in grapes and grape-derived products [3]. Many methods have been used to control *A. carbonarius* and subsequent OTA contamination, including physical, chemical, and biological approaches, with chemical methods being the most prevalent [4]. However, growing concerns regarding food safety necessitate the development of efficient, natural, non-toxic, and harmless chemical preservatives to prevent and control *A. carbonarius* and OTA contamination.

Dimethyl fumarate (DMF) is a chemical compound known for its low toxicity, high antifungal efficiency, and ability to act through both contact and fumigation mechanisms, making it superior to many conventional preservatives [5]. It is commonly used in feed, cosmetics, bread, fish, meat, vegetables, and fruits for mildew prevention, preservation, and as an insect repellent [6]. Despite its effectiveness, the European Union strictly regulates dimethyl fumarate due to health concerns, limiting its concentration to a maximum of 0.1 mg/kg by weight of the product or part of the product [7]. In the field of medicine, dimethyl fumarate was initially approved in the United States in 2013 and approved in Europe in 2014 for the treatment of relapsing–remitting multiple sclerosis and psoriasis [8]. Salvatore et al. [9] found that dimethyl fumarate may help mitigate the inflammatory processes and oxidative reactions associated with diabetic retinopathy, positioning it as a potential candidate drug for the treatment of diabetic retinopathy. Subsequently, Ma et al. [10] investigated the effects of dimethyl fumarate on mycotoxins and animal growth performance. They found that dimethyl fumarate not only detoxified mycotoxins but also promoted a balanced gut microbiome. Their findings offered new insights into the integration of traditional physical methods to remove surface molds with innovative additives, presenting novel strategies for controlling mycotoxins in animal feed.

Although dimethyl fumarate has demonstrated antifungal activity against several postharvest pathogens, including *Aspergillus niger*, *Penicillium* spp., and *Fusarium* spp. [10], the antifungal mechanism of dimethyl fumarate against *A. carbonarius* remains largely unknown. This gap in knowledge highlights the importance of using transcriptomic approaches to explore gene expression changes in fungi under chemical stress. Prior transcriptome studies have revealed key genes and pathways associated with mycotoxin biosynthesis in *Aspergillus ochraceus* [11], oxidative stress response in *Aspergillus flavus* [12], and aflatoxin regulation in *Aspergillus niger* [13]. These examples underscore the utility of transcriptomics in uncovering molecular mechanisms in fungal pathogenicity and stress adaptation.

Building on these previous studies, this study will examine the inhibitory effect of dimethyl fumarate fumigation on the growth of *A. carbonarius*, explore its antifungal mechanism through transcriptomics analysis, and clarify the function of key pathway-related genes. This research aims to provide a scientific foundation for the application of dimethyl fumarate in inhibiting *A. carbonarius*.

## 2. Results

### 2.1. Inhibitory Effect of Dimethyl Fumarate on Mycelial Growth

**Figure 1:**
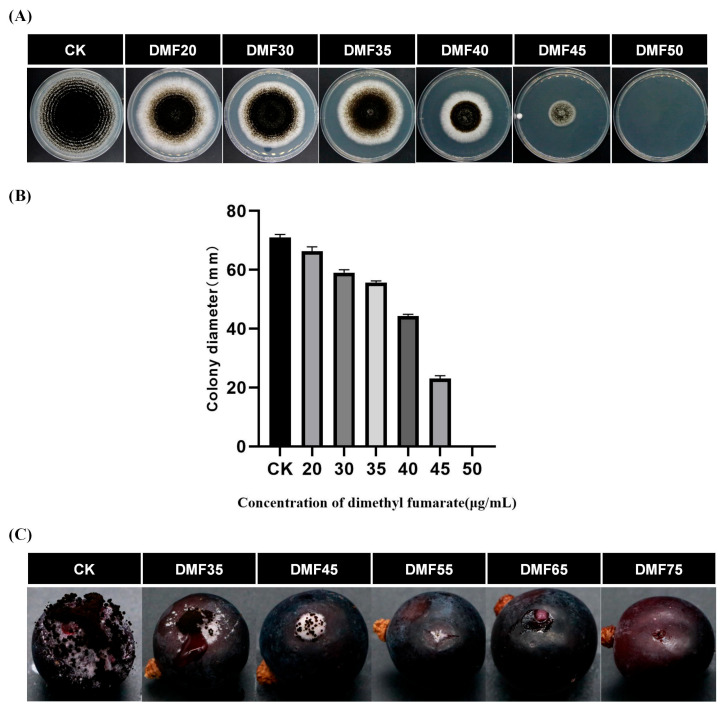
Effect of dimethyl fumarate on the mycelial growth of *A. carbonarius*. (**A**) Fumigation with different concentrations of dimethyl fumarate (0, 20, 30, 35, 40, 45, 50 μg/mL). (**B**) Colony diameter at different concentrations of dimethyl fumarate. (**C**) Inhibition effect of dimethyl fumarate on *A. carbonarius* on grapes. CK = control; DMF35, 45, 55, 65, 75 = treated with 35, 45, 55, 65, 75 µg/mL dimethyl fumarate.

The fumigation of *A. carbonarius* on PDA medium demonstrated a dose-dependent inhibition of mycelial growth with varying concentrations of dimethyl fumarate (Figure 1A). When the concentration of dimethyl fumarate was 40 and 45 μg/mL, the inhibition rate of mycelial growth increased from 38% to 69%. Notably, at a concentration of 50 μg/mL, the mycelial growth was completely inhibited (Figure 1B). No detectable levels of ochratoxin A were found in either the control (CK) or DMF-treated samples.

As shown in Figure 1C, the inhibitory effect of different concentrations of dimethyl fumarate on *A. carbonarius* on grapes also showed a significant dose dependence. However, compared with *A. carbonarius* inoculated on the PDA plates, the dose concentration of dimethyl fumarate required for the complete inhibition of *A. carbonarius* inoculated on grapes is higher. When the concentration of dimethyl fumarate is 75 μg/mL, *A. carbonarius* on grapes can be completely inhibited.

### 2.2. SEM Analysis of Mycelial Morphology

**Figure 2:**
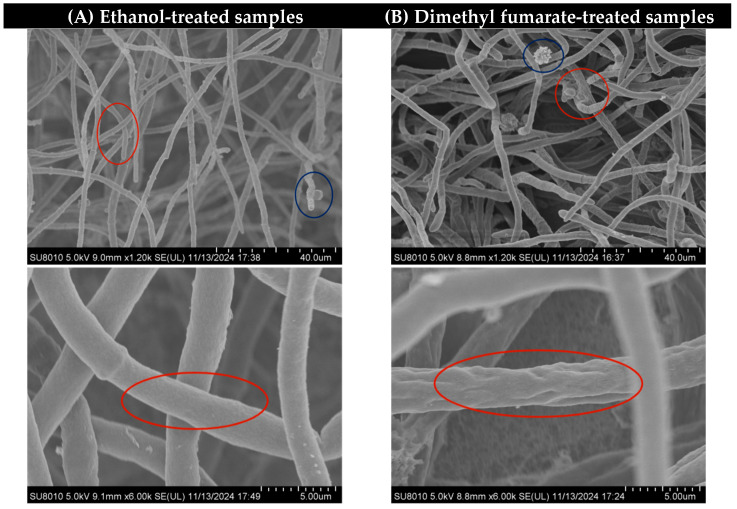
The mycelium morphology of different treatments under the magnification of 1.2 k and 6 k of SEM. The two figures in column (**A**) represent the control samples treated with ethanol, while those in column (**B**) represent samples treated with 35 μg/mL dimethyl fumarate.

As shown in Figure 2, depicting an SEM image at a magnification of 1.2 k, in the red circle, it can be seen that some hyphae treated with dimethyl fumarate were adhered and entangled together, and in the blue circle, it can also be seen that the conidium head was deformed and broken and lost integrity after being treated with dimethyl fumarate. At a higher magnification of 6 k, the mycelium diameter in the ethanol treatment control group appears relatively uniform with a smooth surface. In contrast, the mycelium treated with dimethyl fumarate exhibits significant shrinkage and numerous folds on its surface. Comparing the two sets of images in Figure 2, it can be inferred that the treatment of dimethyl fumarate destroyed the normal growth of *A. carbonarius* mycelia and conidia.

### 2.3. Transcriptomic Analysis

#### 2.3.1. Identification and Screening of DEGs Under Dimethyl Fumarate Treatment

**Figure 3:**
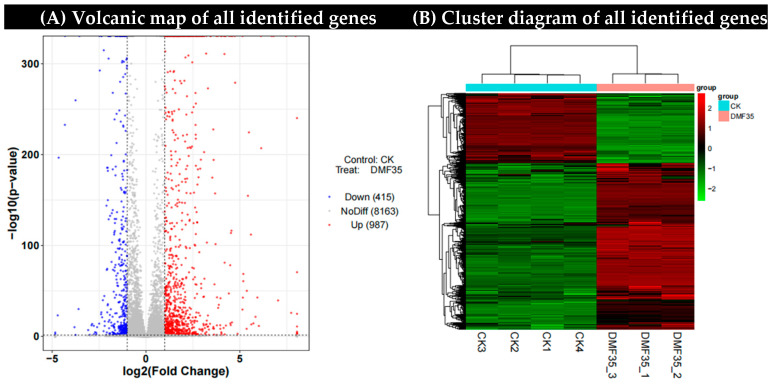
(**A**) Volcanic map of all identified genes. (**B**) The heat map showed the hierarchical clustering of *A. carbonarius* genes under dimethyl fumarate treatment. Each column represents an example. The logarithm is represented by different colors, where red represents a significantly up-regulated gene and green represents a significantly down-regulated gene. CK1, CK2, CK3: Triplicate biological replicates of untreated control; DMF35_1, DMF35_2, DMF35_3: Triplicate biological replicates treated with 35 μg/mL DMF.

In order to characterize the changes in the transcriptome level of *A. carbonarius*, we analyzed the gene expression differences between the control group and the samples treated with 35 μg/mL dimethyl fumarate. A total of 9565 genes were identified (Supplementary Material), and the DEGs were screened according to the expression difference multiple |log2FoldChange| > 1, significant *p*-value < 0.05. A total of 1402 DEGs (FC ≥ 1, *p* < 0.05) were identified, including 987 up-regulated genes and 415 down-regulated genes (Figure 3A). The hierarchical cluster analysis of the DEGs showed that more than 30% of the gene expression was down-regulated in *A. carbonarius* treated with dimethyl fumarate compared to that with the control group (Figure 3B).

Subsequently, the gene expression differences between the control group and the samples treated with 30 and 35 μg/mL dimethyl fumarate were analyzed. It was found that as the concentration of dimethyl fumarate increased, the up-regulated genes and down-regulated genes of *A. carbonarius* increased significantly, and the total number of DEGs also increased.

#### 2.3.2. Functional Classification of DEGs and Pathway Analysis

The GO enrichment analysis results of the DEGs were classified according to molecular function (MF), biological process (BP) and cell component (CC), and the first 10 GO terms with the smallest *p*-value in each GO classification were selected for display.

**Figure 4:**
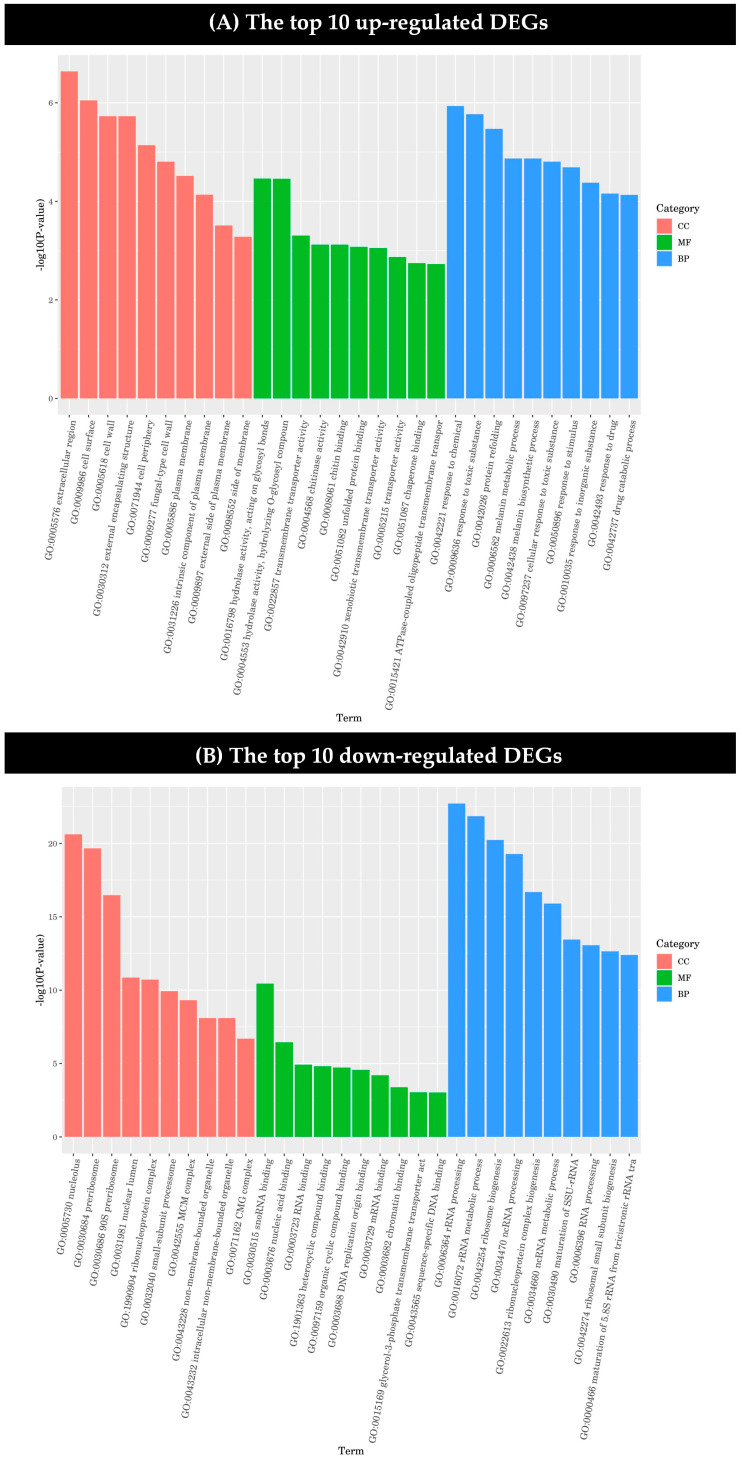
Gene ontology (GO) functional classification of differentially expressed genes (DEGs) was performed. (**A**) The top 10 up-regulated DEGs and (**B**) the top 10 down-regulated DEGs under the treatment of dimethyl fumarate.

Among the up-regulated genes, the genes of the extracellular region, cell surface, cell wall, external encapsulation structure, and cell periphery in cell components were significantly regulated by dimethyl fumarate. The genes coding for hydrolytic enzyme activity acting on the sugar bond in hydrolase and the hydrolysis of the O-sugar component were significantly more affected than by other genes in the molecular function category. In the biological process category, the genes related to the reactions of chemical graphene oxide, the toxic substance graphene oxide, and protein refolding were greatly influenced by dimethyl fumarate (Figure 4A).

As shown in Figure 4, it can be seen that the effect of dimethyl fumarate on the down-regulated genes is significantly higher than that on the up-regulated genes. Among the down-regulated genes, the differential expression of nucleolus, pre-ribosome, and 90S pre-ribosome genes in the cell component category was more significant, and there was also a significant effect on genes involved in snoRNA binding in the molecular function category. It can be seen in Figure 4B that dimethyl fumarate has a more significant effect on the down-regulated DEGs in the biological process category than on those in the cell component and molecular function categories. Among them, rRNA processing, rRNA metabolic process, ribosomal biogenesis, ncRNA processing, ribonucleoprotein complex biogenesis, and ncRNA metabolic process genes were all greatly affected.

**Figure 5:**
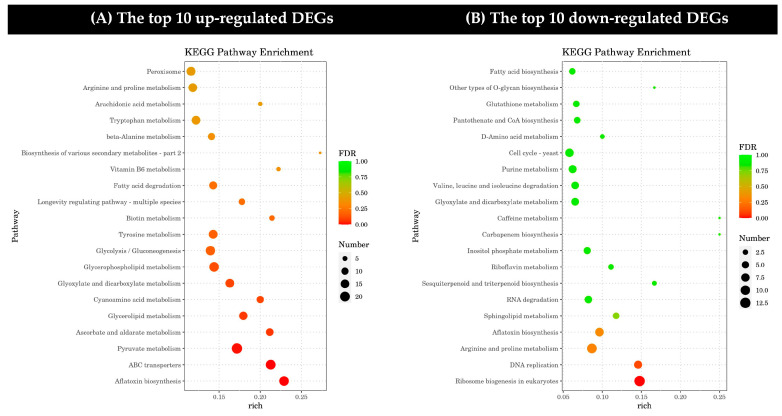
Kyoto Encyclopedia of Genes and Genomes (KEGG) differentially expressed gene pathway enrichment (DEGs). (**A**) The top 10 up-regulated DEGs and (**B**) the top 10 down-regulated DEGs under the treatment of dimethyl fumarate. In each graph, the DEGs are sorted in ascending order of enrichment level.

Through a KEGG pathway analysis, the top 20 up-regulated and down-regulated DEGs were assigned to metabolic pathways (Figure 5A,B). The up-regulated DEGs mainly affected a wide range of biochemical processes in six broad categories: cellular processes, environmental information processing, genetic information processing, metabolism, and organic systems. The down-regulated DEGs mainly affected four broad categories of biochemical processes: cellular processes, genetic information processing, metabolism, and organism systems. In addition, the up-regulated and down-regulated DEGs in the metabolism category were both highly expressed. Among them, genes related to ABC transporters in environmental information processing, aflatoxin biosynthesis, and pyruvate metabolism in metabolism were more abundant in the up-regulated pathway, while genes involved in ribosome biosynthesis and eukaryotic DNA replication in genetic information processing were more significant in the down-regulated pathway.

#### 2.3.3. Functional Analysis of DEGs

In this study, some of the DEGs were considered to be main genes related to cell integrity, fungal development, and stress response. After the dimethyl fumarate treatment, some of the DEGs related to these three aspects showed significant differences, as shown in Table 1. Among them, *ChiB*, *fcyB*, *mok13*, *PCH_Pc12g07500*, and *nvfG* were primarily associated with cell integrity; *LaeA* and *acyN* with fungal development; *abaA* and *wetA* with conidiophore development; and *LUC7* and *gst3* with stress [14]. The up-regulation and down-regulation of these differentially expressed genes have a certain inhibitory effect on the growth and development of *A. carbonarius*.

**Figure 6:**
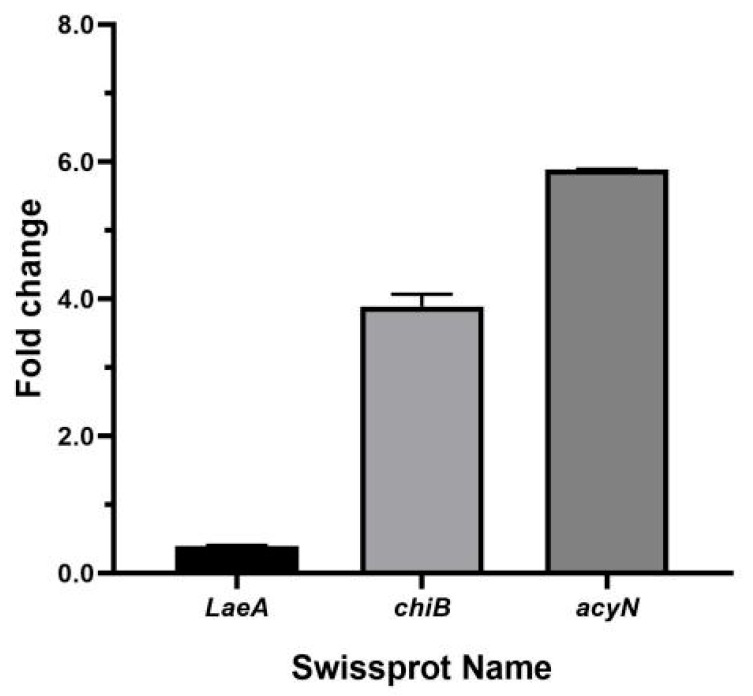
Expression levels of three significantly differentially expressed genes in *A. carbonarius* under 35 μg/mL DMF.

In order to verify the above RNA-Seq data, three significant DEGs, *LaeA*, *chiB*, and *acyN*, were selected and analyzed by RT-qPCR. As shown in Figure 6, the obtained gene expression is consistent with the transcriptome data.

## 3. Discussion

These findings regarding the inhibitory effect of dimethyl fumarate on mycelial growth in this study were consistent with the results of previous studies. Zhang et al. [15] found that dimethyl fumarate had a good inhibitory effect on *Penicillium expansum*, *Rhizopus oryzae*, *Aspergillus niger*, *Mucor racemosus*, *Aspergillus terreus*, and *Penicillium decumbens isolated* from tobacco. Among them, the inhibitory effect of dimethyl fumarate on *P. expansum*, *A. niger*, and *A. terreus* was the strongest, showing complete inhibition at a concentration of 0.4 mg/mL. Billie et al. [16] found that dimethyl fumarate could inhibit the fungal growth of *Aspergillus flavus*, *A. niger*, yeast, and *Staphylococcus aureus*. At a concentration of 500 mg/kg, *A. flavus* and *S. aureus* were completely inhibited, while yeast was completely inhibited at a concentration of 800 mg/kg. In general, the inhibition rate of dimethyl fumarate depends on its concentration. Therefore, dimethyl fumarate has significant antifungal properties and can inhibit the growth of many fungi, including *A. carbonarius*.

Among the genes associated with fungal development, *LaeA* acts as a global regulator of secondary metabolism in filamentous fungi. The deletion of *LaeA* blocked the expression of metabolic gene clusters such as those associated with penicillin, steroid macrocystin, and lovastatin. And the overexpression of *LaeA* enhances the transcription of these genes and promotes the formation of several secondary products [17]. *LaeA* is also directly involved in regulating the transcription of genes in the OTA biosynthetic pathway and can affect conidia formation and conidial germination [18]. In the present study, only the *LaeA* gene was significantly down-regulated among the main genes related to fungal development. This result is consistent with the results shown in Figure 1. The conidia number of *A. carbonarius* decreased with the increase in dimethyl fumarate concentration. Tsunematsu et al. [19] found that knocking out *LaeA* in the model mushroom *Stropharia cinereus* unexpectedly up-regulated the biosynthesis of a novel siderophore-associated protein, while the deletion of cpf1, which encodes a non-ribosomal peptide synthetase involved in methemoglobin biosynthesis, led to growth defects and abolished fruiting body formation. This demonstrates that *LaeA*, although best known for controlling secondary metabolism, can also act as a repressor of specific metabolites involved in essential cellular processes such as virulence. Contrastingly, in our experiments with *Aspergillus carbonarius*, the down-regulation of *LaeA* led to inhibited fungal growth, impaired development, and disrupted secondary metabolism. This apparent contradiction implies that *LaeA*’s regulatory role may be context-dependent, potentially exerting positive or negative control over metabolic pathways depending on the fungal species or environmental conditions.

As shown in Table 1, the secondary metabolic regulator *LaeA* gene was significantly down-regulated, while the non-ribosomal peptide synthase *acyN* gene was significantly up-regulated. The *acyN* gene is also involved in the biosynthesis of secondary metabolites, metabolic pathways, and the biosynthesis of ubiquinone and other terpenoid quinones. Previous studies have reported that many siderophores are synthesized by non-ribosomal peptide synthetases [20]. Therefore, we hypothesize that the up-regulated NRPS gene *acyN* may be involved in siderophore-mediated iron metabolism, thereby accelerating the metabolism of iron and reducing iron availability. Iron is an indispensable cofactor for all microbial life [21], so the reduction of iron may contribute to the inhibition of fungal growth.

DEGs related to cell integrity also play a certain inhibitory effect on the growth of *A. carbonarius*. The endochitinase *chiB* gene showed the highest up-regulation among these DEGs related to cell integrity. Studies have shown that the endochitinase *chiB* gene belongs to the antifungal gene category, and inhibits mycelial growth by inducing the deformation of the mycelium [22]. In this study, treatment with dimethyl fumarate led to significant up-regulation of the *chiB* gene, and the mycelia of *A. carbonarius* were deformed and shrunk. Previous studies have also shown that *A. carbonarius* treated with Rhodiola rosea essential oil showed mycelial aggregation, wrinkling, emptying, collapsing, and becoming equal in size [23]. These results suggest that destroying the integrity of the membrane and reducing the rigidity of the cell wall by up-regulation of the endochitinase *chiB* gene may contribute to the inhibition of *A. carbonarius* growth by dimethyl fumarate.

**Figure 7:**
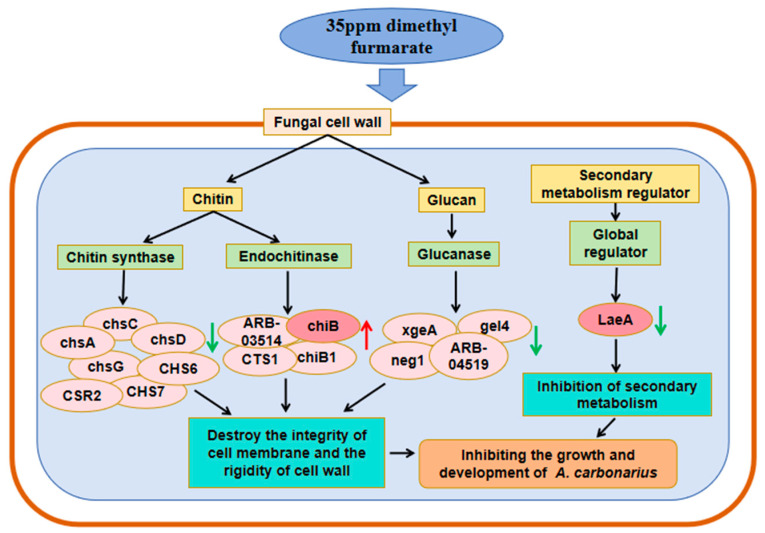
Based on the transcriptome data obtained in this study, a graphical model of the mechanism of dimethyl fumarate affecting the growth and development of *A. carbonarius* was established. Red arrows represent up-regulated genes, while green arrows represent down-regulated genes.

The fungal cell wall is mainly composed of chitin and glucan [24]. As shown in Figure 7, in addition to the negative feedback regulation of four endochitinase genes including *chiB*, seven chitin synthase genes such as *chsA*, *chsC*, and *CHS6*, and four glucanase genes including xgeA and *neg1* were all down-regulated after treatment with dimethyl fumarate. The down-regulation might affect the composition of the cell wall of *A. carbonarius*, reduce the rigidity of the cell wall, and then inhibit the fungal growth.

Taken together, following treatment with dimethyl fumarate, *A. carbonarius* exhibited significant differential expression of genes related to cell integrity and development. The genes related to fungal growth and development genes were down-regulated, especially *LaeA*. In addition, genes related to cell wall synthesis were down-regulated, especially *chsA*, *chsC*, *CHS6*, *xgeA*, and *neg1*; meanwhile, the genes related to cell wall degradation were up-regulated, especially *chiB*.

## 4. Conclusions

This study indicated that dimethyl fumarate could efficiently inhibit the growth of *A. carbonarius* in a dose-dependent manner. The antifungal mechanism was further investigated by a transcriptomic analysis and SEM observations. The results suggest that dimethyl fumarate exerts an inhibitory effect by destroying the cell integrity, inhibiting mycelial growth and development, altering the conidiophore structure, and disturbing the secondary mechanism. Genes related to fungal growth and development were down-regulated, genes related to cell wall synthesis were down-regulated, genes related to asexual development were up-regulated, and genes related to cell wall degradation were up-regulated. This provides a theoretical basis for the application of dimethyl fumarate in the inhibition of *A. carbonarius* in the feed and fruit industry.

## 5. Materials and Methods

### 5.1. Chemicals, Fungal Strain, and Growth Conditions

Dimethyl fumarate (purity: 97%, CAS number: 624-49-7) was purchased from Shanghai Yuanye Biotechnology Co., Ltd. (Shanghai, China). *A. carbonarius* was provided by Ren Xueyan Laboratory, School of Food Engineering and Nutrition Science, Shaanxi Normal University (Xian, Shannxi, China). *A. carbonarius* was inoculated at the center of potato dextrose agar (PDA) plates and cultured at 28 °C in darkness for 10 days [25]. Then, the conidia were collected by washing with 0.1% Tween-80 solution, and the conidia concentration was adjusted to 106 conidia/mL, as determined using a hemocytometer [26].

### 5.2. Antifungal Activity of Dimethyl Fumarate

To prepare a 16.67 mg/mL solution of dimethyl fumarate, 0.5 g dimethyl fumarate was dissolved in 30 mL ethanol, leveraging its high solubility in DMF versus water. This original solution was then diluted to the final concentrations of 90%, 80%, 70%, and 60%. Qualitative filter paper was punched into 6 mm diameter disks, which were subsequently placed in Petri dishes and autoclaved at 121 °C for 15 min for future use.

Regarding the in vitro filter paper fumigation experiment, some modifications were made [27]. A total of 2.5 μL of *A. carbonarius* conidia suspension was added dropwise to the center of the PDA plate. After the conidia suspension was dried, a 6 mm filter paper was placed on the cover of the Petri dish. The prepared five concentrations of dimethyl fumarate solution were added dropwise to 150 μL on the filter paper, so that the concentration of dimethyl fumarate in the space of the Petri dish reached 30, 35, 40, 45, and 50 μg/mL. The plate with 150 μL of ethanol was used as a control for fumigation experiments. Then, the plate was placed in the dark at 28 °C for 12 days, and the colony diameter was measured. The inhibition of dimethyl fumarate on *A. carbonarius* was observed. Each treatment was repeated three times.

The minimum inhibitory concentration (MIC) [28,29] was defined as the minimum concentration of dimethyl fumarate that inhibits visible mycelial growth. The growth inhibition rate was calculated as follows: I (%) = (Dc − Dt) ×100/Dc. I: Inhibition rate of mycelial growth; Dc: colony diameter of control group; Dt: colony diameter of inhibition group.

Summer Black grapes (Vitis Vinfera “Summer Black”) were purchased from a supermarket in Honghe, Yunnan, China, and shipped to the laboratory. Each grape was cut off the vine, and the fruits with uniform size and maturity without any mechanical damage were selected. The fruit and a small box with a volume of 25 mL were soaked in 95% ethanol and sterilized by ultraviolet irradiation. Then, on an ultra-clean bench, the sterilized filter paper was placed in the small box with tweezers, and the previously prepared dimethyl fumarate solution of different concentrations was added dropwise to make the concentration of dimethyl fumarate in the space in the box reach 35, 45, 55, 65, and 75 μg/mL. The sterilized toothpick was used to puncture the center of the grapes, and 5 μL of *A. carbonarius* conidia suspension (10^6^ conidia/mL) was used to inoculate them. The grapes were placed with the wound facing upward in the box, which was sealed, and placed in the dark at 28 °C for 7 days to observe the lesions on the grapes.

### 5.3. OTA Detection

After shaking for 2 h in a rotating shaker, the supernatant was filtered through a 0.22 μm filter into a brown vial. All extracts were stored at −20 °C until high-performance liquid chromatography (HPLC) analysis. The HPLC equipment consisted of an Agilent 1260 series system (Agilent Technologies, Inc., Santa Clara, CA, USA) with a fluorescence detector and an autosampler. Analysis was carried out in isocratic mode with a mobile phase of acetonitrile (ACN)/water/acetic acid (99:99:2 *v*/*v*/*v*) at a flow rate of 1 mL min^−1^. The injection volume was 20 μL. Fluorescence detection (FLD) was performed at an excitation wavelength of 330 nm and an emission wavelength of 460 nm, using a C18 column (Agilent, 150 mm × 4.6 mm, 5 μm internal diameter). Pure OTA standard was obtained from Sigma-Aldrich (St. Louis, MO, USA).

### 5.4. Scanning Electron Microscopy (SEM) Analysis

According to previous studies, SEM was carried out [30], and sterilized glass slides were inserted on a PDA plate with a suspension of *A. carbonarius* conidia [31], treated with dimethyl fumarate (35 μg/mL), and treated with the same amount of ethanol as a control. After 12 days of culture, the mycelium block at the edge of the fungus colony was cut and put into the fixative solution, followed by a series of pretreatments, and its morphology was observed under an SEM.

### 5.5. Total RNA Isolation, cDNA Library Construction, and Sequencing

The *A. carbonarius* isolate used in this study has been deposited in GenBank under accession number GCA_001990825.1. The mycelia were cultured with ethanol, 30 μg/mL dimethyl fumarate, and 35 μg/mL dimethyl fumarate for transcriptome sequencing. Ethanol-treated cultures were used as the solvent control for the transcriptome analysis, since dimethyl fumarate is ethanol-soluble. RNA extraction, cDNA library construction, and RNA-seq analysis were performed by Huatai Yikang (Beijing) Biotechnology Co., Ltd. (HTHealth) (Beijing, China). Raw reads were assessed using FastQC (version 0.11.9), trimmed with Trimmomatic, aligned to the *A. carbonarius* genome using HISAT2 (version 2.2.1), and counted using HTSeq (version 0.12.4). Differential expression analysis was conducted with DESeq2 (version 1.36.0).

According to the manufacturer instructions, total RNA was isolated and purified with TRIzol reagent, and each experiment was performed three times. The quantity, purity, and integrity of RNA were evaluated, with an RIN value > 7.0, and identified by denaturing agarose gel electrophoresis. Then, the mRNA with polyA structure in the total RNA was enriched by Oligo (dT) magnetic beads, and the RNA was broken into fragments of about 300 bp by ion interruption. The first strand of cDNA was synthesized by using RNA as template, 6-base random primers, and reverse transcriptase, and the second strand of cDNA was synthesized by using the first strand of cDNA as template.

After the library construction was completed, the library fragments were enriched by PCR amplification, and then the library was selected according to the fragment size. The library size was 450 bp. Then, the quality of the library was tested using an Agilent 2100 Bioanalyzer, and the total concentration of the library and the effective concentration of the library were detected. Then, according to the effective concentration of the library and the amount of data required by the library, the libraries containing different Index sequences are proportionally mixed. The mixed library was uniformly diluted to 2 nM, and the single-strand library was formed by alkali denaturation.

After RNA extraction, purification, and library construction, the libraries were sequenced by Next-Generation Sequencing (NGS) on the Illumina sequencing platform.

### 5.6. Database Search and Analysis

Firstly, the original Raw Data was filtered, and the high-quality Clean Data obtained after filtering was compared with the reference genome of *A. carbonarius* (GCA_001990825.1_Aspca3_genomic.fna). According to the comparison results, the expression of each gene was calculated. And further expression difference analysis and functional enrichment analysis of differentially expressed genes were carried out.

### 5.7. Differential Expression Analysis

DESeq was used to analyze the differences in gene expression, and the conditions for screening differentially expressed genes (DEGs) were as follows: expression difference multiple |log2FoldChange| > 1, significant *p*-value < 0.05. Then, the R language package ggplot2 (version 3.4.0) was used to draw the volcanic map of the DEGs, showing the gene distribution, gene expression fold difference, and significant results. The R package pheatmap (version 1.0.12) was used to perform two-way cluster analysis on the union of differential genes and samples of all comparison groups. According to the expression level of the same gene in different samples and the expression patterns of different genes in the same sample, clustering was performed. The Euclidean method was used to calculate the distance, and the hierarchical clustering longest distance method was used for clustering.

### 5.8. Functional Enrichment Analysis of Differentially Expressed Genes

Firstly, topGO was used for GO enrichment analysis, and the gene list and number of each term were calculated by using the DEGs annotated by GO term. Then, the *p*-value was calculated using the hypergeometric distribution method (the standard of significant enrichment was *p*-value < 0.05), and the GO term with significant enrichment of DEGs was found compared with the whole genome background, so as to determine the main biological functions of DEGs. KEGG enrichment analysis was performed using the same method. According to the results of KEGG enrichment, the top 30 pathways with the smallest *p*-value, that is, the most significant enrichment, were selected for display, and the degree of enrichment was measured using the Rich factor, FDR value, and the number of genes enriched in this pathway.

### 5.9. Gene Expression Analysis Performed with RT-qPCR

DEGs with significant up-regulation and down-regulation were selected for transcriptional verification. Total RNA was extracted from dimethyl fumarate-treated samples using an RNA kit, and then was reverse-transcribed into cDNA by reverse transcriptase. Primers were designed according to the gene transcription sequence of *A. carbonarius* (Table 2), and then RT-qPCR analysis was performed. The relative transcription level was determined using the 2−ΔΔCt method [32].

### 5.10. Data Analysis

In this study, each experiment was repeated three times, and all experimental data were expressed as mean ± standard deviation (SD), and *p*-value < 0.05 was considered statistically significant. All statistical analyses were performed using SPSS statisticsl software (version 22.0; IBM Corp., Armonk, NY, USA). The statistical graph in this paper was made using GraphPad Prism software (version 8.0.2; GraphPad Software, San Diego, CA, USA).

## Figures and Tables

**Table 1:** Significantly differentially expressed genes between dimethyl fumarate treatment and control in *Aspergillus carbonarius*.

	Gene ID	Swissprot Name	log2FoldChange	*p*-Value	Annotated Gene Function
Cell integrity	ASPCADRAFT_203630	*chiB*	2.137	9.288 × 10^−185^	Endochitinase
	ASPCADRAFT_125551	*chiB1*	1.657	1.139 × 10^−13^	
	ASPCADRAFT_208876	*ARB_03514*	1.561	1.837 × 10^−17^	
	ASPCADRAFT_205337	*CTS1*	0.955	4.009 × 10^−22^	
	ASPCADRAFT_210467	*xgeA*	−1.438	1.383 × 10^−106^	Glucanase
	ASPCADRAFT_208658	*gel4*	−0.717	1.000× 10^−3^	
	ASPCADRAFT_212042	*neg1*	−0.456	3.283 × 10^−6^	
	ASPCADRAFT_206132	*ARB_04519*	−0.517	2.478 × 10^−55^	
	ASPCADRAFT_206390	*fcyB*	1.489	1.020 × 10^−53^	Purine-cytosine permease
	ASPCADRAFT_135110	*PCH_Pc12g07500*	1.468	4.070 × 10^−26^	Mutanase
	ASPCADRAFT_503657	*mok13*	1.053	2.498 × 10^−49^	Cell wall alpha-1,3-glucan synthase
	ASPCADRAFT_203143	*nvfG*	−1.923	3.034 × 10^−24^	Ketoreductase
	ASPCADRAFT_211936	*chsD*	−0.867	1.417 × 10^−168^	Chitin synthase
	ASPCADRAFT_208758	*chsG*	−0.419	8.630 × 10^−57^	
	ASPCADRAFT_209480	*CSR2*	−0.519	5.100 × 10^−32^	
	ASPCADRAFT_203230	*CHS6*	−0.255	2.991 × 10^−22^	
	ASPCADRAFT_208304	*chsC*	−0.422	2.749 × 10^−14^	
	ASPCADRAFT_211141	*chsA*	−0.384	7.000× 10^−3^	
	ASPCADRAFT_159307	*chs7*	−0.273	0.844	
Development	ASPCADRAFT_208824	*acyN*	2.697	0.000	Nonribosomal peptide synthetase
	ASPCADRAFT_5941	*LaeA*	−1.119	2.691 × 10^−10^	Secondary metabolism regulator
	ASPCADRAFT_212444	*brlA*	2.279	3.074 × 10^−199^	regulator of conidiophore development
	ASPCADRAFT_169479	*wetA*	1.091	2.241 × 10^−6^	Developmental regulatory protein
Stress response	ASPCADRAFT_207672	*LUC7*	2.683	0	Glutathione S-transferase-like protein
	ASPCADRAFT_174114	*gst3*	1.837	4.817 × 10^−38^	Glutathione S-transferase 3
	ASPCADRAFT_131005	*gst3*	1.345	6.182 × 10^−5^	Glutathione S-transferase 3

**Table 2:** The upstream primers and downstream primers of RT-qPCR for *laeA*, *chiB*, and *acyN* genes.

Gene ID	Swissprot Name	Upstream Primer	Downstream Primer
ASPCADRAFT_5941	*laeA*	taatgctcgcatcctcgacc	tgagatcccaggaatcctctc
ASPCADRAFT_203630	*chiB*	ctgatccaacatcgacacgc	caccatgatctcctacgacactg
ASPCADRAFT_208824	*acyN*	cattctcttccgccatcatcg	cccattggtttctgtcggtagg

## Data Availability

The original contributions presented in this study are included in the article/Supplementary Material. Further inquiries can be directed to the corresponding author.

## Supplementary Materials

The following supporting information can be downloaded at https://www.mdpi.com/article/10.3390/toxins17070339/s1: A portion of the DEGs are presented in the results, while the remaining data are provided in the Supplementary Materials.

## Abbreviations

The following abbreviations are used in this manuscript:

DEGs	Differentially expressed genes
OTA	Ochratoxin A
HPLC	High-performance liquid chromatography
